# A human single-neuron dataset for face perception

**DOI:** 10.1038/s41597-022-01482-4

**Published:** 2022-06-25

**Authors:** Runnan Cao, Chujun Lin, Nicholas J. Brandmeir, Shuo Wang

**Affiliations:** 1 Lane Department of Computer Science and Electrical Engineering, West Virginia University, Morgantown, WV 26506 USA; 2 Department of Psychological and Brain Sciences, Dartmouth College, Hanover, NH 03755 USA; 3 Department of Neurosurgery, West Virginia University, Morgantown, WV 26506 USA; 4 Department of Radiology, Washington University in St. Louis, St. Louis, MO 63110 USA

**Keywords:** Perception, Social neuroscience

## Abstract

The human amygdala and hippocampus have long been associated with face perception. Here, we present a dataset of single-neuron activity in the human amygdala and hippocampus during face perception. We recorded 2082 neurons from the human amygdala and hippocampus when neurosurgical patients with intractable epilepsy performed a one-back task using natural face stimuli, which mimics natural face perception. Specifically, our data include (1) single-neuron activity from the amygdala (996 neurons) and hippocampus (1086 neurons), (2) eye movements (gaze position and pupil), (3) psychological assessment of the patients, and (4) social trait judgment ratings from a subset of patients and a large sample of participants from the general population. Together, our comprehensive dataset with a large population of neurons can facilitate multifaceted investigation of face perception with the highest spatial and temporal resolution currently available in humans.

## Abstract (summary)

**Table:** 

Measurement(s)	Single-neuron activity • Eye movement • Social trait judgment ratings
Technology Type(s)	Human single-neuron recordings • Eye tracking • Rating tasks

## Background & Summary

The human amygdala and hippocampus demonstrate important functional roles in face perception^1–4^. They have been implicated in multiple functions related to face processing. First, neurons in the amygdala and hippocampus encode face identities^5,6^ as well as the conceptual relationship between face identities^7,8^. Our recent data have shown that neurons in the amygdala and hippocampus encode visually similar face identities and embody feature-based coding of faces^9^, a plausible mechanism that may explain how identity coding emerges from the amygdala and hippocampus. Second, neurons in the amygdala and hippocampus demonstrate category-selective response to faces^10,11^ and such response can be modulated by attention^11^. Third, neurons in the amygdala and hippocampus encode salient facial features such as the eyes and mouth, which may in turn play important roles in face recognition and judgment^12^. Lastly, neurons in the amygdala and hippocampus are not only selective to emotion categories (especially amygdala neurons)^13–15^ but also encode a comprehensive social trait space that underlies spontaneous first impressions^16^.

However, there is a lack of a publicly available dataset to comprehensively understand these face perception processes at the neuronal level in the human brain. Here, we present a large dataset including not only human single-neuron activity but also simultaneously recorded eye movements during face perception. We used natural celebrity faces^17^ and employed a one-back task, which mimics natural, real-life conditions when people perceive faces and forms first impressions, and avoids potential biases from focusing on a particular facial feature (e.g., compared to asking participants to judge facial trustworthiness). This task has been shown to be very effective to study neural face representation in humans^9,18^. We recorded from 2082 neurons in total from 12 neurosurgical patients (40 sessions), with 996 neurons from the amygdala and 1086 neurons from the hippocampus (Table 1). We also acquired social trait judgment ratings from a subset of patients (*n* = 6) as well as from a large sample of participants from the general population (*n* = 439). Together, our direct recordings from the human brain at the single-neuron level will not only provide a key missing link between human neuroimaging and single-neuron recordings in non-human animals but also facilitate multifaceted investigations of the neural and computational mechanisms underlying face perception.

**Table 1:** Patients and sessions.

ID	Age	Sex	Race	Epilepsy diagnosis	Number of sessions	Number of neurons	Eye tracking	Social trait	AQ	SRS	BDI
p6WV	33	F	Caucasian	Left posterior neocortical extratemporal/parietal	2	95	2	50	12	17	0
p7WV	28	F	Caucasian	Right mesial temporal	4	148	3	150	21	n/a	23
p9WV	42	M	Caucasian	Left frontal	4	138	4	0	n/a	n/a	14
p10WV	47	F	Caucasian	Right mesial temporal and neocortical temporal	3	116	1	0	29	n/a	19
p11WV	33	F	Caucasian	Right mesial temporal and extratemporal	5	105	3	250	11	46	7
p13WV	41	M	Caucasian	Left hippocampal	1	5	1	0	20	13	1
p14WV	26	M	Caucasian	Bilateral amygdylar/hippocampal	4	173	3	500	11	10	4
p15WV	37	F	Caucasian	Left amygdylar/hippocampal	2	61	2	0	11	n/a	2
p16WV	29	F	Caucasian	Right temporal neocortex	6	548	2	500	10	38	5
p18WV	53	F	Caucasian	Left temporal	4	303	4	0	22	n/a	13
p19WV	49	M	Caucasian	Right mesial temporal onset	2	53	2	0	33	n/a	n.a
p20WV	31	F	Caucasian	Left temporal	3	337	2	500	21	48	15

Eye tracking: the number of sessions with eye tracking data available. Although all sessions had simultaneous eye tracking, we only included sessions that had more than 10 fixations onto each facial region of interest (ROI; some sessions had a substantial amount of missing eye tracking data). Social trait: the number of faces that patients provided social trait judgment ratings (note that for each face, judgments were provided for all 8 social traits). AQ: Autism Spectrum Quotient. SRS: Social Responsiveness Scale-Adult Research Version. BDI: Beck’s Depression Inventory. n/a: not available.

## Methods

The detailed methods have been described in our previous studies^9,12,16^. Below, we provide an overview of our methods.

### Patients

There were 40 sessions with 12 patients in total (Table 1). All patients provided written informed consent using procedures approved by the Institutional Review Board of West Virginia University (WVU). Psychological assessment including Autism Spectrum Quotient (AQ), Social Responsiveness Scale (SRS; Adult Research Version), and Beck’s Depression Inventory (BDI) was performed for each patient (Table 1).

### Stimuli

We used faces of celebrities from the CelebA dataset^17^. We selected 50 identities with 10 images for each identity, totaling 500 face images. Our stimuli included both genders (33 of the 50 identities were male) and multiple races (40 identities were Caucasian, 9 identities were African-American, and 1 identity was biracial), allowing us to investigate the dependency of identity coding on gender and race^9^. We used the same stimuli for all patients. All images had the same resolution, and the faces had a similar size and position in the images^17^. We further showed in our previous study that image resolution, brightness, background, and other low-level image features could not explain face identity coding^9^.

We asked patients to indicate whether they were familiar with each identity in a follow-up survey. Nine out of 12 patients provided familiarity/recognition judgment, and within this data, 40.7% ± 21.5% (mean ± SD across patients) of the face identities were judged as familiar (i.e., patients recognized the identity of the face). Because we focused on the coding of *identities* rather than *concepts*, we did not expect the patients to be familiar with all identities and we did not inquire about their depth of knowledge regarding the identities, simply whether they recognized them (i.e., face familiarity). On the other hand, although prior knowledge of the familiar faces may influence face perception (in particular social trait judgment^19–21^), neural face coding could be replicated using unfamiliar faces and even FaceGen model faces^9^.

### Experimental procedure

We used a one-back task. In each trial, a single face was presented at the center of the screen for a fixed duration of 1 s, with uniformly jittered inter-stimulus-interval (ISI) of 0.5–0.75 s. Each image subtended a visual angle of approximately 10°. Patients pressed a button if the present face image was *identical* to the immediately previous image. 9% of trials were one-back repetitions. Each face was shown once unless repeated in one-back trials; and we excluded responses from one-back trials to have an equal number of responses for each face. This task kept patients attending to the faces, but avoided potential biases from focusing on a particular facial feature (e.g., compared to asking patients to judge a particular facial feature). The order of faces was randomized for each patient. This task procedure has been shown to be effective to study face representation in humans^18^.

### Electrophysiology

**Fig. 1:**
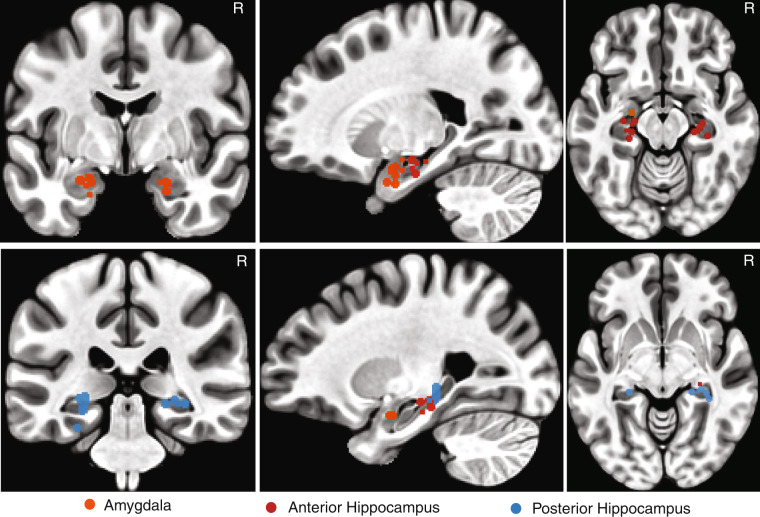
Electrode locations in the MNI space. The recording sites of each patient were estimated according to the pre-implantation T1-weighted MRI and post-implantation CT. All sagittal slices are from the left hemisphere. R: right.

We recorded from implanted depth electrodes in the amygdala and hippocampus from patients with pharmacologically intractable epilepsy. Recording locations (Fig. 1 and Table 2) were estimated based on post-operative CT scans (orientation = axial, slice number = 195, 0.485 × 485 mm in-plane resolution, 1 mm thick, tube current = 320 mA, matrix size = 512 × 512) that were performed after implantation of the electrodes. These scans were registered to pre-operative T1-weighted MRI scans (orientation = axial, slice number = 156, 0.976 × 0.976 mm in-plane, 1 mm thick, TR = 8248 ms, TE = 3.548 ms, matrix size = 256 × 256, flip angle = 12°) to allow patient-specific localization. We used the function ‘align_epi_anat.py’ in AFNI to align the CT images to the MRI T1 images. The T1 images were then normalized to the MNI152 standard template to obtain a transformation matrix. We further applied the transformation matrix to the CT images to align them to the MNI space. Finally, we manually located each electrode position and derived the MNI coordinates for each recording location.

**Table 2:** Estimated recording locations for each patient.

ID	Left Amygdala	Right Amygdala	Left Anterior Hippocampus	Right Anterior Hippocampus	Left Posterior Hippocampus	Right Posterior Hippocampus
p6WV	19, 3, −21	n.a.	29, 16, −13	n.a.	25, 32, −6	n.a.
p7WV	21, 6, −18	n.a.	23, 24, −15	n.a.	25, 32, −3	n.a.
p9WV	20, 1, −27	n.a.	23, 19, −16	n.a.	26, 31, −11	n.a.
p10WV	23, 10, −14	−25, −1, −22	26, 28, −11	n.a.	28, 32, −4	n.a.
p11WV	n.a.	−19, 2, −21	n.a.	−29, 18, −15	n.a.	−29, 30, −7
p13WV	20, 4, −19	n.a.	25, 29, −15	n.a.	29, 25, −18	n.a.
p14WV	20, 6, −28	−23, 2, −22	20, 18, −22	−36, 23, −18	29, 32, −21	−35, 37, −7
p15WV	22, 6, −23	n.a.	26, 23, −12	−34, 19, −18	n.a.	n.a.
p16WV	23, 10, −20	−22, 5, −21	27, 23, −12	−27, 21, −13	26, 32, −9	−27, 31, −5
p18WV	25, 7, −20	−23, 6, −21	19, 16, −19	−33, 27, −13	n.a.	−33, 33, −7
p19WV	n.a.	−22, 5, −26	28, 24, −18	−26, 24, −16	n.a.	−22, 32, −7
p20WV	25, 5, −20	n.a.	21, 16, −14	−23, 24, −15	24, 28, −11	n.a.

Shown are radiological coordinates (also known as RAI: right-anterior-inferior) in the MNI space. n.a.: not available (no electrodes implanted at this location).

At each site, we recorded from eight 40 μm microwires inserted into a clinical electrode as described previously^22,23^. Bipolar wide-band recordings (0.1–9000 Hz), using one of the eight microwires as reference, were sampled at 32 kHz and stored continuously for off-line analysis with a Neuralynx system. The raw signal was filtered with a zero-phase lag 300–3000 Hz bandpass filter and spikes were sorted using a semi-automatic template matching algorithm as described previously^24^.

Units were carefully isolated and recording and spike sorting quality were assessed quantitatively. Isolation distance was calculated based on^25,26^. If a cluster contains *n*_*C*_ cluster spikes, the isolation distance of the cluster is the *D*^2^ value of the *n*_*C*_^th^ closest noise spike. Isolation distance is therefore the radius of the smallest ellipsoid from the cluster center containing all of the cluster spikes and an equal number of noise spikes. As such, isolation distance estimates how distant the cluster spikes are from the other spikes recorded on the same electrode. Isolation distance is not defined for cases in which the number of cluster spikes is greater than the number of noise spikes. Consistent with our previous studies^9,11,12,14–16^, only single units with an average firing rate of at least 0.15 Hz throughout the entire task were considered.

### Eye tracking

Patients were recorded with a remote non-invasive infrared Eyelink 1000 system (SR Research, Canada). One of the eyes was tracked at 500 Hz. The eye tracker was calibrated with the built-in 9-point grid method at the beginning of each block. Fixation extraction was carried out using software supplied with the Eyelink eye tracking system. Saccade detection required a deflection of greater than 0.1°, with a minimum velocity of 30°/s and a minimum acceleration of 8000°/s^2^, maintained for at least 4 ms. Fixations were defined as the complement of a saccade, i.e., periods without saccades. Analysis of the eye movement record was carried out off-line after completion of the experiments.

To quantitatively compare the fixations within certain parts of the face, we defined three regions of interest (ROIs): eyes, mouth, and nose. For each image, we first identified the 5 landmarks (center of left eye, center of right eye, nasal tip, left corner of mouth, and right corner of mouth) using the multitask cascaded convolutional networks (MTCNN)^27^. We then drew a rectangular bounding box for each ROI. For the eye ROI, the left edge of the bounding box was 18 pixels left to the left eye landmark, the right edge was 18 pixels right to the right eye landmark, the upper edge was 13 pixels above the mean eye landmarks, and the lower edge was 8 pixels below the mean eye landmarks. For the mouth ROI, the left edge of the bounding box was 8 pixels left to the left mouth landmark, the right edge was 10 pixels right to the right mouth landmark, the upper edge was 10 pixels above the mean mouth landmarks, and the lower edge was 12 pixels below the mean mouth landmarks. For the nose ROI, the left edge, right edge, upper edge, and lower edge of the bounding box were 20 pixels left, 20 pixels right, 13 pixels above, and 5 pixels below the nose landmark, respectively. Lastly, we manually checked and adjusted the positions of the ROIs (especially for profile faces) to ensure that all ROIs well captured the relevant facial parts. The fixation density was calculated for each participant during the entire 1 second stimulus period, and was normalized within each participant. Fixation locations were smoothed using a 2D Gaussian kernel (30 pixel by 30 pixel) with a standard deviation of 3 pixels.

All participants had normal or corrected-to-normal visual acuity. We excluded 11 sessions that had fewer than 10 fixations onto each ROI due to a substantial amount of missing eye tracking data. The excluded sessions came from 6 patients (Table 1). It is worth noting that only eye tracking analysis was excluded from the discarded sessions but identity coding and social trait judgment analyses used data from all sessions.

### Online rating of social traits

Participants were asked to rate the faces on eight social traits using a 7-point Likert scale through an online rating task. The social traits included *warm, critical, competent, practical, feminine, strong, youthful*, and *charismatic*. These social traits were well validated in a previous study^28^. Based on that study, these eight social traits represented the four core psychological dimensions of comprehensive trait judgments of faces (warmth, competence, femininity, and youth). The four comprehensive dimensions were derived from an exploratory factor analysis, and they were weakly correlated. Specifically, each comprehensive dimension was represented by two social traits in our study: *warm* and *critical* for warmth, *competent* and *practical* for competence, *feminine* and *strong* for femininity, and *youthful* and *charismatic* for youth. One trait was the dimension’s label (e.g., *warm* is the label of the warmth dimension), and the other trait had high factor loading on that dimension according to the factor analysis that derived the four comprehensive trait dimensions (e.g., *critical* had high factor loading on the warmth dimension).

Patients completed the social trait rating task online after they were discharged from the hospital following surgery to treat intractable epilepsy. Six patients completed the rating task and provided ratings for 1 to 10 photos per identity per social trait. Participants from the general population completed the rating task using the Prolific online research platform. Participants who were fluent in English were included. We divided the experiment into 10 modules, with each module containing one face image randomly selected per face identity (totaling 50 face images per module), and we required participants to rate each face within a module on 8 social traits (e.g., competence; rated in blocks). We collected data from 43.9 participants on average per module. All face images in the stimulus set (500 in total) were rated on all 8 social traits, totaling 175,600 ratings across all participants and modules.

We applied the following three exclusion criteria: (1) Trial-wise exclusion: we excluded trials with reaction times shorter than 100 ms or longer than 5000 ms. (2) Block/trait-wise exclusion: we excluded the entire block per participant if more than 30% of the trials were excluded from the block per (1) above, or if there were fewer than 3 different rating values in the block (this suggests that the participant may not have used the rating scale properly). (3) Participant-wise exclusion: we excluded a participant if more than 3 blocks were excluded from the participant per (2) above. Based on the above criteria, 24 (5.47%) participants and 7882 (4.49%) trials were excluded from further analysis. Specifically, we excluded 25 modules for *warm*, 36 modules for *critical*, 32 modules for *competent*, 36 modules for *practical*, 49 modules for *feminine*, 29 modules for *strong*, 26 modules for *youthful*, and 30 modules for *charismatic*.

Inter-rater consistency of each trait was estimated using the intraclass correlation coefficient (ICC; two-way random-effects model for the consistency of mean ratings)^29^ and the Spearman’s correlation coefficient (*ρ*). The ICC and Spearman’s *ρ* were computed between raters for each trait in each module and then averaged across modules per trait. The ICC was calculated using Matlab implementation written by Arash Salarian (2020) (https://www.mathworks.com/matlabcentral/fileexchange/22099-intraclass-correlation-coefficient-icc). The Spearman’s *ρ* was computed between each pair of raters and then averaged across all pairs of raters.

## Data Records

The data^30^ consist of stimuli used in the experiments, behavior, events, eye movement, neuronal spikes, and social trait judgment ratings. All data (except stimuli) are stored in MATLAB.mat files. Different parts of the data are organized in different directories and described in detail below. A detailed description of variables and usage can be found in ‘readme.rtf’ in each data directory.

### Stimuli and ROIs

The stimuli used in the present study are located in the ‘/Stimuli/CelebA/’ directory. The mapping between the filename of the face image and the index in the behavioral data (see below) can be found in the file ‘/Stimuli/FaceImageIndex.csv’. The name of the identity for each face image is stored in the variable ‘im_code’ in ‘Stimuli/CelebA_Image_Code_new.mat’. We asked patients whether they could recognize each face identity, and the labels for the recognized identities can be found in the file ‘/Stimuli/CelebA_Recognition.mat’.

Coordinates for ROIs are located in ‘/ET/Code/CelebA_ROI.mat’. The variables ‘ROI_E’, ‘ROI_M’, and ‘ROI_N’ contain the coordinates for the eyes, mouth, and nose, respectively, for each face image. We use the indices 1, 2, 3, and 0 to denote the ROI for eyes, mouth, nose, and other parts, respectively.

### Behavior

Behavioral data for each patient is stored in the ‘behaviorData/p*WV/CelebA/Sess*/’ directory (* indicates the session number). The timing parameters for each trial can be found in the variable ‘iT’. Stimulus presentation order is in the variable ‘code’, which contains the index for each face image. Indices for the repeated (i.e., one-back) trials are stored in the variable ‘back_id’. If the patient responded (i.e., pressed the space bar) in the one-back trial, the value is 1 for this trial in the variable ‘vResp’; otherwise, the value is 0. Response time is stored in the variable ‘RT’.

### Events

Timestamps of events (i.e., stimulus onsets) are stored in the ‘/Events Files/’ directly. There is one file for each session. The variable ‘periods’ contains 3 columns. The first column denotes trial indices, the second column denotes the timestamps (in μs) corresponding to 500 ms before stimulus onset, and the third column denotes timestamps corresponding to 1500 ms after stimulus onset.

### Neuronal spikes

Neuronal spikes were sorted and extracted using an established procedure^24^. We combined neurons from all sessions into a single file (‘/Single Neuron/Data/Spikes.mat’). For each neuron, the timestamps (in μs), session ID, channel ID, cluster ID (i.e., randomly assigned neuron ID within a channel), and recording brain area are stored in the variables ‘timestampsOfCellAll’, ‘vCell’, ‘vCh’, ‘vClusterID’, and ‘areaCell’, respectively (note that these variables were all matched). There are three variables that describe spike sorting quality. The variable ‘IsolDist’ shows the isolation distance value for each cluster (i.e., a single neuron; see Methods). The variable ‘statsSNR’ contains 6 columns: 1—session ID, 2—channel ID, 3—cluster ID, 4—the average signal-to-noise ratio (SNR), 5—inter-spike intervals (ISI) that are below 3 ms, and 6—peak SNR. The variable ‘statsProjAll’ contains 5 columns: 1—session ID, 2—channel ID, 3 and 4—IDs for a cluster pair, and 5—pairwise distance between two clusters.

### Eye tracking data

Eye tracking data are located in the folder ‘/ET/Data/’ and there is one file for each session. The eye tracking data for each trial were aligned to the image coordinates and then were extracted and stored in a matrix format in the variable ‘eyeMatrix’. In each matrix, each row refers to a single fixation and the following saccade and the columns (27 in total) refer to the following attributes: 1—trial index, 2—fixation index, 3—fixation coordinate in horizontal direction (aligned to image coordinates), 4—fixation coordinate in vertical direction, 5—fixation start time (in ms, relative to image onset), 6—fixation end time, 7—face image index, 8—ROI that the fixation falls in, 9—fixation time in each ROI, 10—empty (not used), 11— average pupil size, 12—fixation duration, 13—saccade index, 14—saccade starting position in horizontal direction, 15—saccade starting position in vertical direction, 16—saccade ending position in horizontal direction, 17—saccade ending position in vertical direction, 18—saccade starting time, 19—saccade ending time, 20—saccade duration, 21—saccade distance (in degrees of visual angle), 22—saccade peak velocity (in visual degrees per second), 23—saccade starting ROI (1: eyes, 2: mouth, 3: nose, 0: other), 24—saccade ending ROI, 25—serial order of saccade with starting position in this ROI (e.g., ‘2’ means the second saccade in this trial with the starting position in this ROI), 26—serial order of saccade with ending position in this ROI, and 27—saccade direction (e.g., a number ‘12’ denotes saccade from the eyes to the mouth; note that digit 4 rather than 0 is used here to denote other parts).

### Social trait judgment ratings

Rating data are located in the folder ‘/Social Rating/Data/’. The data for patients are stored in ‘CelebA_PatientRating.mat’ and the data for online participants are stored in ‘CelebA_ProlificRating.mat’. In each data file, there are three variables: ‘traits’ refers to the trait names, ‘SubIDPerModule’ refers to the included subject ID for each module, and ‘data’ refers to the rating data. In the variable ‘data’, each row refers to a face, and the columns (7 in total) refer to the following attributes: 1—ratings of all valid trials (all excluded trials were replaced with NaN; see Methods for exclusion criteria), 2—ratings of all trials (without exclusion), 3—response time of all valid trials in ms (response time of excluded trials were replaced with NaN), 4—response time of all trials (without exclusion), 5—module index, 6—name of the face image, and 7—identity label. Demographics of patients can be found in Table 1. Demographics of online participants can be found in ‘Demographic_Prolific.mat’.

## Technical Validation

### Quality metrics for spike sorting

**Fig. 2:**
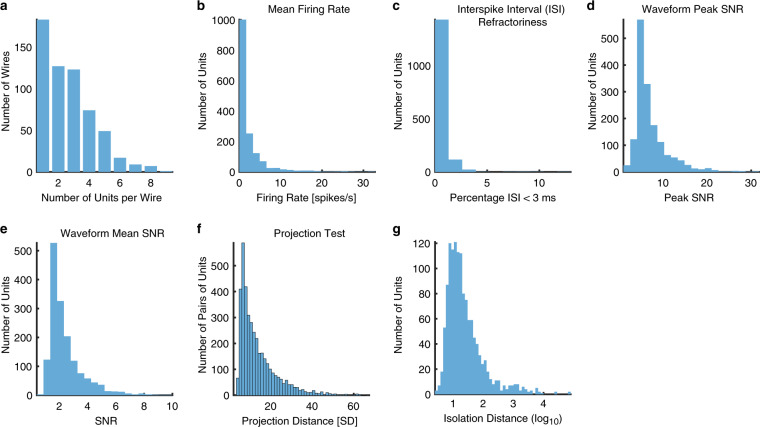
Assessment of recording and spike sorting quality. (**a**) Histogram of the number of units identified on each active wire (only wires with at least one unit identified are counted). The average yield per wire with at least one unit was 2.54 ± 1.62 (mean ± SD). (**b**) Histogram of mean firing rates. (**c**) Histogram of proportion of inter-spike intervals (ISIs) which are shorter than 3 ms. The large majority of clusters had less than 0.5% of such short ISIs. (**d**) Histogram of the signal-to-noise ratio (SNR) of the mean waveform peak of each unit. (**e**) Histogram of the SNR of the entire waveform of all units. (**f**) Pairwise distance between all possible pairs of units on all wires where more than 1 cluster was isolated. Distances are expressed in units of standard deviation (SD) after normalizing the data such that the distribution of waveforms around their mean is equal to 1. (**g**) Isolation distance of all units for which this metric was defined (*n* = 1577, median = 1.23).

We recorded from the human amygdala and hippocampus (Fig. 1). 1577 units had an overall firing rate greater than 0.15 Hz and we restricted our analysis to this subset of units. We first assessed the quality of our recordings and showed that the quality was comparable to previous studies^11,14,15,31^. Specifically, the number of units recorded per wire was 2.67 ± 1.63 (mean ± SD; in wires with at least one unit; Fig. 2a). The mean firing rate was 2.42 ± 3.87 Hz (Fig. 2b). The percentage of inter-spike intervals (ISIs) below 3 ms was 0.51% ± 0.77% (Fig. 2c). The ratio between the peak amplitude of the mean waveform of each cluster and the standard deviation of the noise (peak signal-to-noise ratio [SNR]) was 7.20 ± 3.98 (Fig. 2d). The mean SNR was 2.42 ± 1.24 (Fig. 2e). The pairwise distance (projection test) between all possible pairs of units on all wires with more than one cluster was isolated and the mean was 13.19 ± 9.47 (Fig. 2f). The median isolation distance was 17.00 (Fig. 2g).

### Eye tracking

**Fig. 3:**
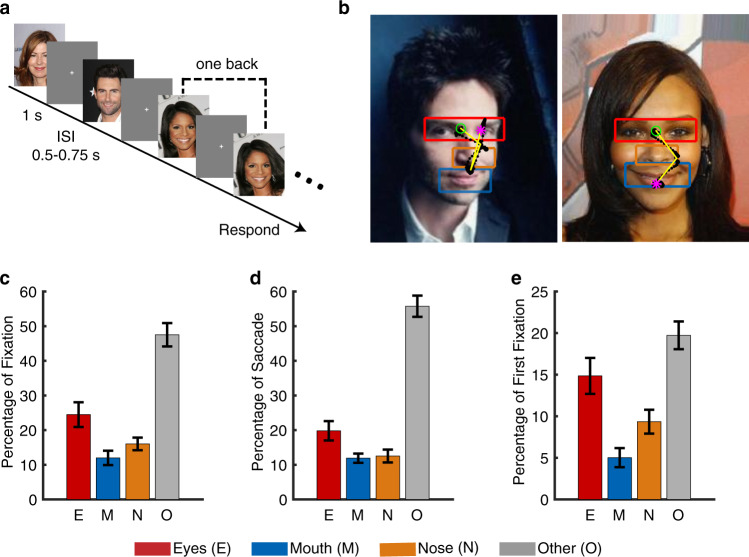
Assessment of eye tracking quality. (**a**) Task. We employed a one-back task in which patients responded whenever an identical famous face was repeated. Each face was presented for 1 s, followed by a jittered inter-stimulus-interval (ISI) of 0.5 to 0.75 seconds. Each image subtended a visual angle of approximately 10°. (**b**) Sample stimuli. Regions of interest (ROIs) were detected using computer vision (not shown to participants). Each yellow dot represents a fixation. Green circle: first fixation. Magenta asterisk: last fixation. Yellow line: saccades. Black dot: raw gaze position. (**c**) Percentage of fixation for each ROI. (**d**) Percentage of saccade to each ROI. (**e**) Percentage of first fixation onto each ROI. Error bars denote ±SEM across sessions. E: eyes. M: mouth. N: nose. O: other (all other parts of the image, including hair, neck, background, etc.).

We next assessed the quality of our eye tracking. We recorded eye movements when patients viewed 500 natural face images of 50 celebrities (10 different photos per identity; Fig. 3a,b). We found that on average, 24.50% ± 18.84% of fixations were on the eyes and 11.98% ± 11.01% of fixations were on the mouth (Fig. 3b,c), consistent with previous studies^32,33^. Furthermore, we found that participants had 19.80% ± 14.78% of saccades to the eyes and 11.90% ± 7.04% of saccades to the mouth (Fig. 3b,d). Among the first fixations, 14.86% ± 11.42% of fixations were on the eyes and 5.02% ± 6.08% of fixations were on the mouth (Fig. 3e).

### Social trait judgment

**Fig. 4:**
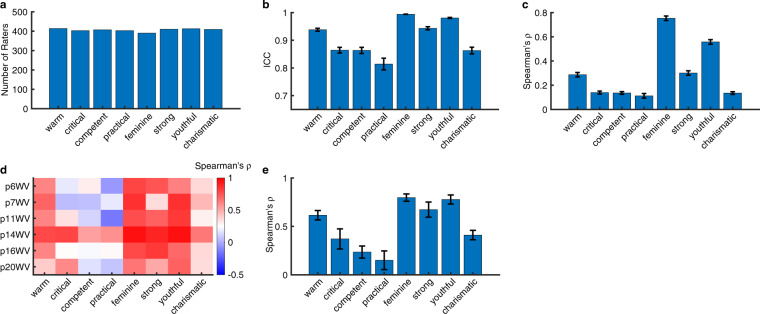
Assessment of social trait ratings. (**a**) Number of online raters per trait. (**b**,**c**) Inter-rater consistency. Inter-rater consistency of each trait was estimated using (**b**) the intraclass correlation coefficient (ICC)^29^ and (**c**) the Spearman’s correlation coefficient (*ρ*). Inter-rater consistency was first calculated between raters and averaged within each module, and then averaged across modules. Error bars denote ±SEM across modules. (**b**) Intraclass correlation coefficient (ICC). (**c**) Spearman’s ρ. (**d**,**e**) Correlation of social trait ratings between neurosurgical patients and online participants (using consensus ratings). (**d**) Individual patient (y-axis). Color coding shows the Spearman’s correlation coefficient ρ. (**e**) Average across patients. Error bars denote ±SEM across patients.

We next assessed social trait judgment ratings. We acquired ratings on 8 traits from 6 patients (325 ± 201.87 [mean ± SD] faces were rated for each trait; Table 1) and a large population of participants recruited via an online platform (406.13 ± 7.68 [mean ± SD] raters per trait; Fig. 4a). The inter-rater consistency of these ratings from online participants (Fig. 4b,c; see Methods; all ICCs > 0.8) was comparable to the established study (i.e., ICCs greater than 0.75 for most of the traits)^28^. We also showed that ratings from patients were generally consistent with the consensus ratings from the online sample (Fig. 4d,e).

### Summary of three categories of neurons

We identified three categories of neurons:

1. To select *identity neurons*, we first used a one-way ANOVA to identify neurons with a significantly unequal response to different identities (P < 0.05) in a window 250–1250 ms following stimulus onset. We next imposed an additional criterion to identify the selective identities: the neural response of an identity was 2 standard deviations (SD) above the mean of neural responses from all identities. If a neuron satisfied both criteria, it was defined as an *identity neuron*. We found 155 identity neurons (9.83%, binomial P = 1.67 × 10^−15^; see Fig. 5a,b for examples), consistent with prior recordings from the human MTL^5,7,8^.

**Fig. 5:**
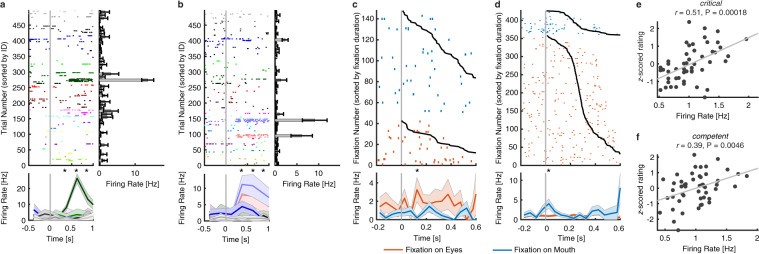
Summary of three categories of neurons. (**a**,**b**) Identity neurons. Shown are neuronal responses to 500 faces (50 identities). Trials are aligned to face stimulus onset (gray line) and are grouped by individual identity. Asterisk indicates a significant difference between face identities in that bin (P < 0.05, one-way ANOVA, after Bonferroni correction for multiple comparisons; bin size = 250 ms). Shaded area denotes ±SEM across trials. Error bars denote ±SEM across faces. (**c**,**d**) Eye-mouth neurons. (**c**) An example eyes-preferring neuron. (**d**) An example mouth-preferring neuron. Fixations are sorted by fixation duration (black line shows start of the next saccade). Fixation onset is t = 0. Asterisk indicates a significant difference between fixations on the eyes and mouth in that bin (P < 0.05, two-tailed *t*-test, after Bonferroni correction for multiple comparisons; bin size = 50 ms). Shaded area denotes ±SEM across fixations. Note that the selection of feature-selective neurons was based on the entire time window. (**e**,**f**) Social-trait neurons. Example neurons that showed a significant correlation between the mean normalized firing rate and the mean *z*-scored rating for (**e**) *critical* and (**f**) *competent*. Each dot represents a face identity, and the gray line denotes the linear fit.
2. To select *eye-mouth neurons*, we aligned neuronal responses at fixation onset and used the mean firing rate in a time window starting 200 ms before fixation onset and ending 200 ms after fixation offset (next saccade onset) to calculate statistics (two-tailed *t*-test, P < 0.05). We identified 164/1085 neurons (15.12%; binomial P < 10^−20^) that had a response differing significantly between fixations on the eyes vs. the mouth and these neurons were defined as *eye-mouth neurons*. We identified two types of such eye-mouth neurons: one type had a greater response to the eyes relative to the mouth (“eyes-preferring”; 102/164 neurons [62.20%]; see Fig. 5c for an example) and the second type had a greater response to the mouth relative to the eyes (“mouth-preferring”; 62/164 neurons [37.80%]; see Fig. 5d for an example).
3. To select *social-trait neurons*, we aligned trials to stimulus onset and used the mean firing rate in a time window 250 ms to 1250 ms after stimulus onset as the response to each face. If a neuron showed a significant correlation with one of the eight social traits (Pearson correlation between the mean normalized firing rate and the mean *z*-scored social trait ratings across 50 identities), it was defined as a *social-trait neuron* (see Fig. 5e,f for examples). In total, we found 113 social-trait neurons.

## Usage Notes

Code for preprocessing each part of the data is located in the corresponding directory ‘*/Code/’ (e.g., to extract neuron firing rate, see ‘/SingleNeuron/Code/SU_getFiringRate.m’). Demonstration code for analyzing each part of the data is located in the ‘/Analysis/’ directory. ‘plotSortingQuality.m’ performs analysis and plotting of spike sorting quality. ‘*NeuronSelection.m’ (* denotes the neuron category) performs statistics and neuron selection. ‘plotRaster*.m’ (* denotes the neuron category) plots activity of example neurons. Variables that need to be adjusted are explained at the beginning of each code. Functions that are common to all analyses are located in the ‘/tools/’ directory.

## Data Availability

The source code is included as part of the dataset^30^. All code is implemented using MATLAB (MathWorks Inc).
